# Supplementary material for: Comparative study on photocatalytic activity of transition metals (Ag and Ni)-doped ZnO nanomaterials synthesized via sol–gel method
Source: R Soc Open Sci. 2020 Feb 26;7(2):191590. doi: 10.1098/rsos.191590 (PMC7062069; doi:10.1098/rsos.191590)
Supplement: Table S1 [file rsos191590supp1.pdf]

Table S1 The morphology, dimension (length and diameter), band gap energy, textural properties and photocatalytic performance of undoped, Ag- and Ni/ZnO (NA= not applicable).

Sample	Morphology	Average length, L (nm)	Average diameter, D (nm)	Band Gap (eV)	$a_{s,BET}$ (m^2g^{-1})	Total pore volume (cm^3g^{-1})	Average pore diameter (nm)	Percentage degradation, (%) after 160 minutes	Photodegradation rate constant, (k, min^{-1})	R^2
Undoped ZnO	Mixture of long nanorods and spherical shapes	242.06	66.01	3.29	11.671	0.1618	55.448	77.66	0.0088	0.9824
1% Ag/ZnO	Mixture of long nanorods and spherical shapes	253.87	65.65	3.32	10.818	0.1641	60.694	98.65	0.0237	0.9408
3% Ag/ZnO	Mixture of long nanorods and spherical shapes	241.21	72.37	3.32	10.003	0.1405	56.163	98.00	0.0206	0.9074
5% Ag/ZnO	Mixture of long nanorods and spherical shapes	275.93	76.06	3.33	10.175	0.1339	52.656	99.93	0.0394	0.9112
7% Ag/ZnO	Mixture of long nanorods and spherical shapes	212.48	76.11	3.33	10.718	0.1688	62.993	98.59	0.0245	0.9714
10% Ag/ZnO	Mixture of long nanorods and spherical shapes	209.95	76.13	3.32	9.7801	0.1363	55.736	97.47	0.0233	0.9624

1% Ni/ZnO	Mixture of long nanorods and spherical shapes	215.63	68.53	3.30	11.970	0.1730	57.828	31.68	0.0029	0.7301
3% Ni/ZnO	Mixture of short nanorods and spherical shapes	185.76	68.02	3.32	12.284	0.1744	56.786	11.12	0.0007	0.9633
5% Ni/ZnO	Mixture of short nanorods and spherical shapes	185.12	66.98	3.33	13.626	0.1898	55.707	15.07	0.001	0.9997
7% Ni/ZnO	Mixture of short nanorods and spherical shapes	151.86	63.27	3.34	13.800	0.1825	51.385	12.11	0.0007	0.8559
10% Ni/ZnO	Spherical shapes	NA	96.15	3.35	14.207	0.1958	56.754	13.44	0.001	0.9702
