# Supplementary material for: Comparative study on photocatalytic activity of transition metals (Ag and Ni)-doped ZnO nanomaterials synthesized via sol–gel method
Source: R Soc Open Sci. 2020 Feb 26;7(2):191590. doi: 10.1098/rsos.191590 (PMC7062069; doi:10.1098/rsos.191590)
Supplement: Supplementary Figures [file rsos191590supp2.pdf]

Supplementary Materials

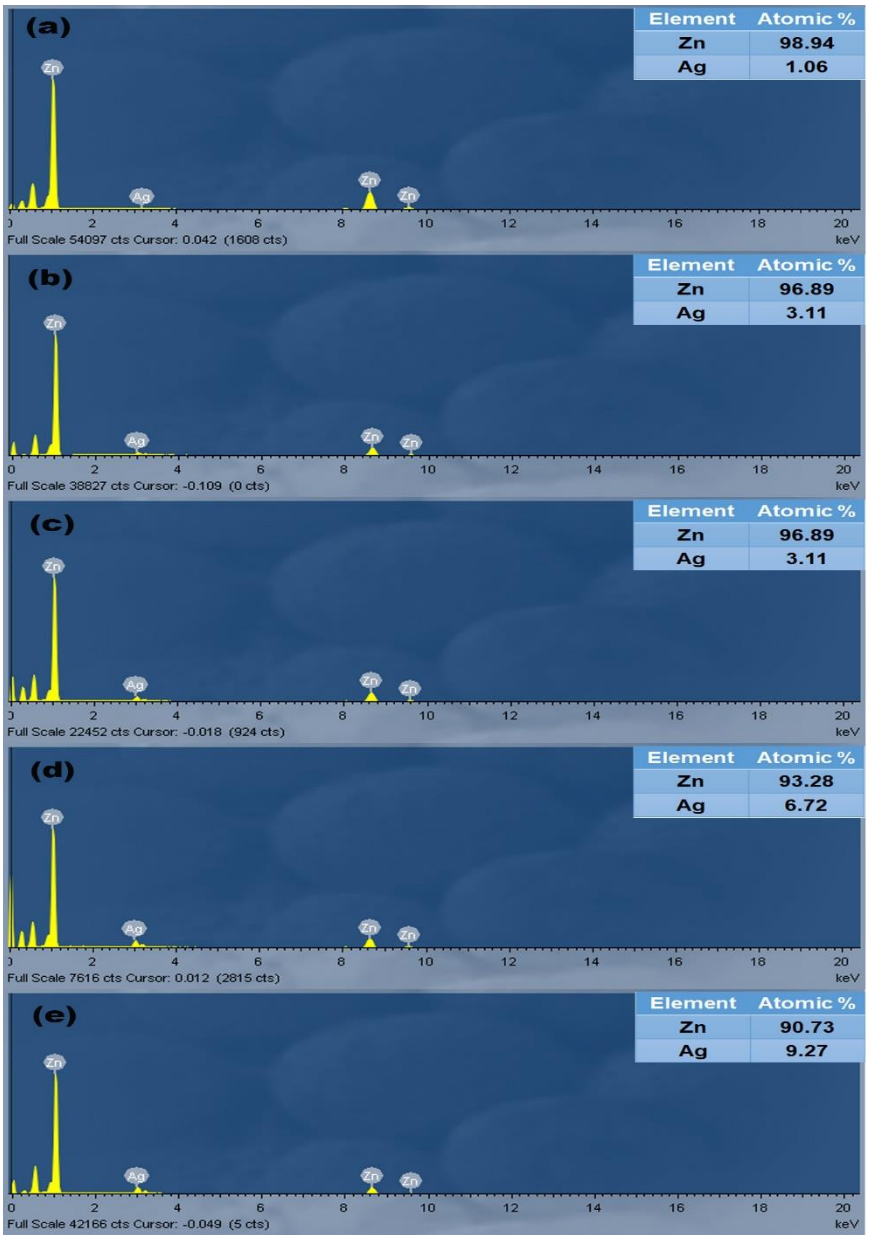

Figure S1. Elemental spectra and composition of **(a)** 1% Ag/ZnO **(b)** 3% Ag/ZnO **(c)** 5% Ag/ZnO **(d)** 7% Ag/ZnO and **(e)** 10% Ag/ZnO nanomaterials.

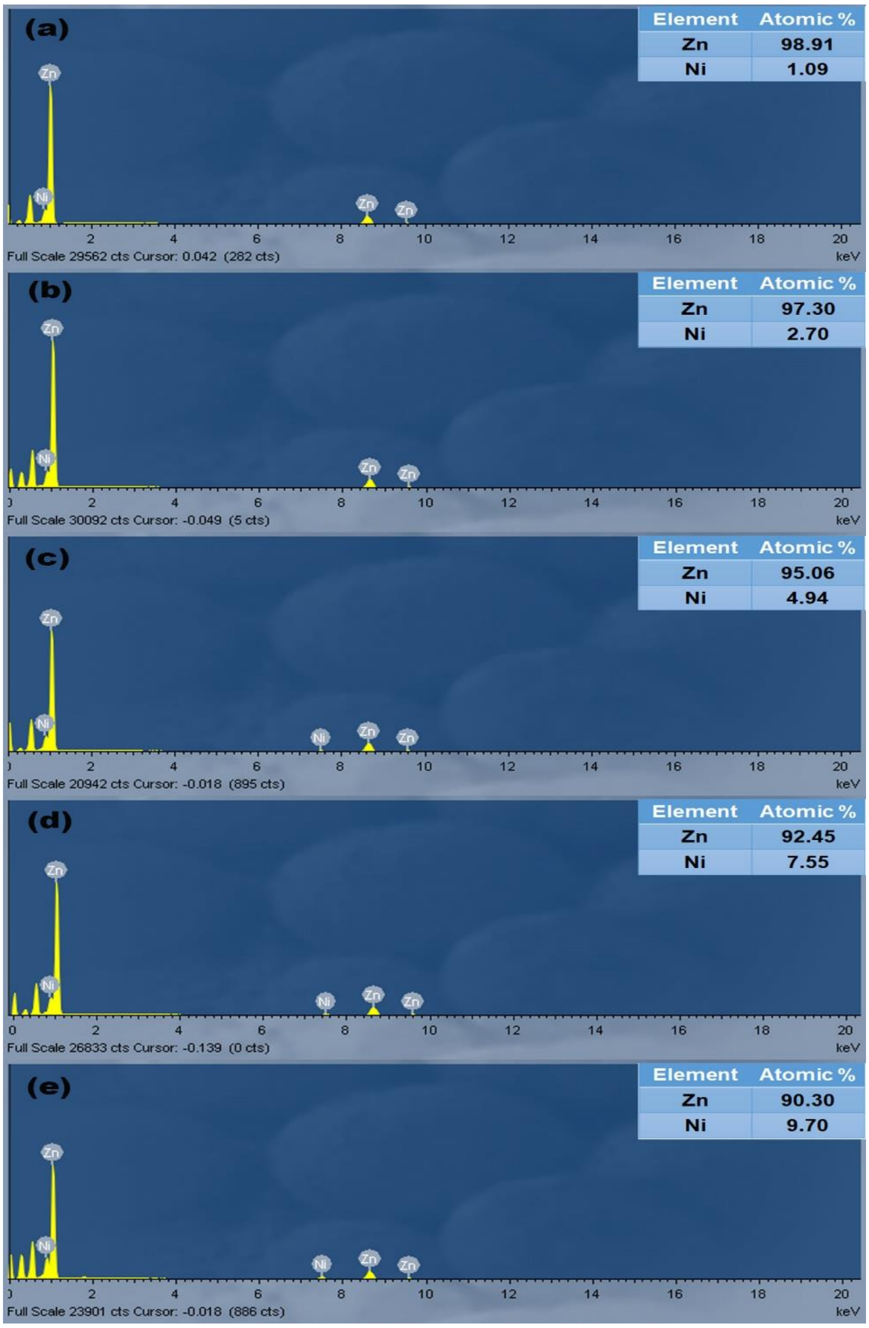

Figure S2. Elemental spectra and composition of **(a)** 1% Ni/ZnO **(b)** 3% Ni/ZnO **(c)** 5% Ni/ZnO **(d)** 7% Ni/ZnO and **(e)** 10% Ni/ZnO nanomaterials.

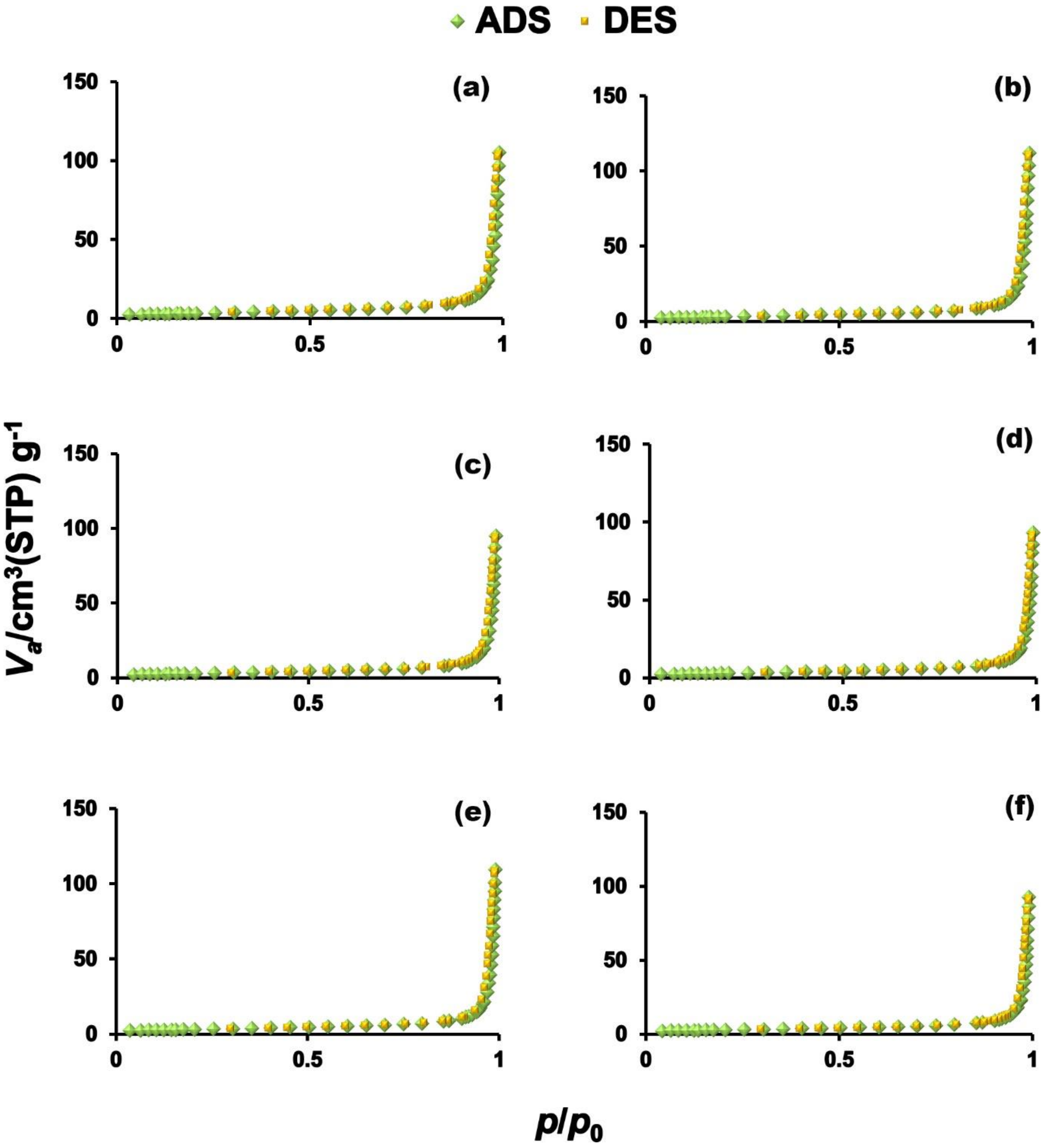

Figure S3. N_2 adsorption-desorption isotherms of **(a)** undoped ZnO **(b)** 1% Ag/ZnO **(c)** 3% Ag/ZnO **(d)** 5% Ag/ZnO **(e)** 7% Ag/ZnO and (**f)** 10% Ag/ZnO nanomaterials.

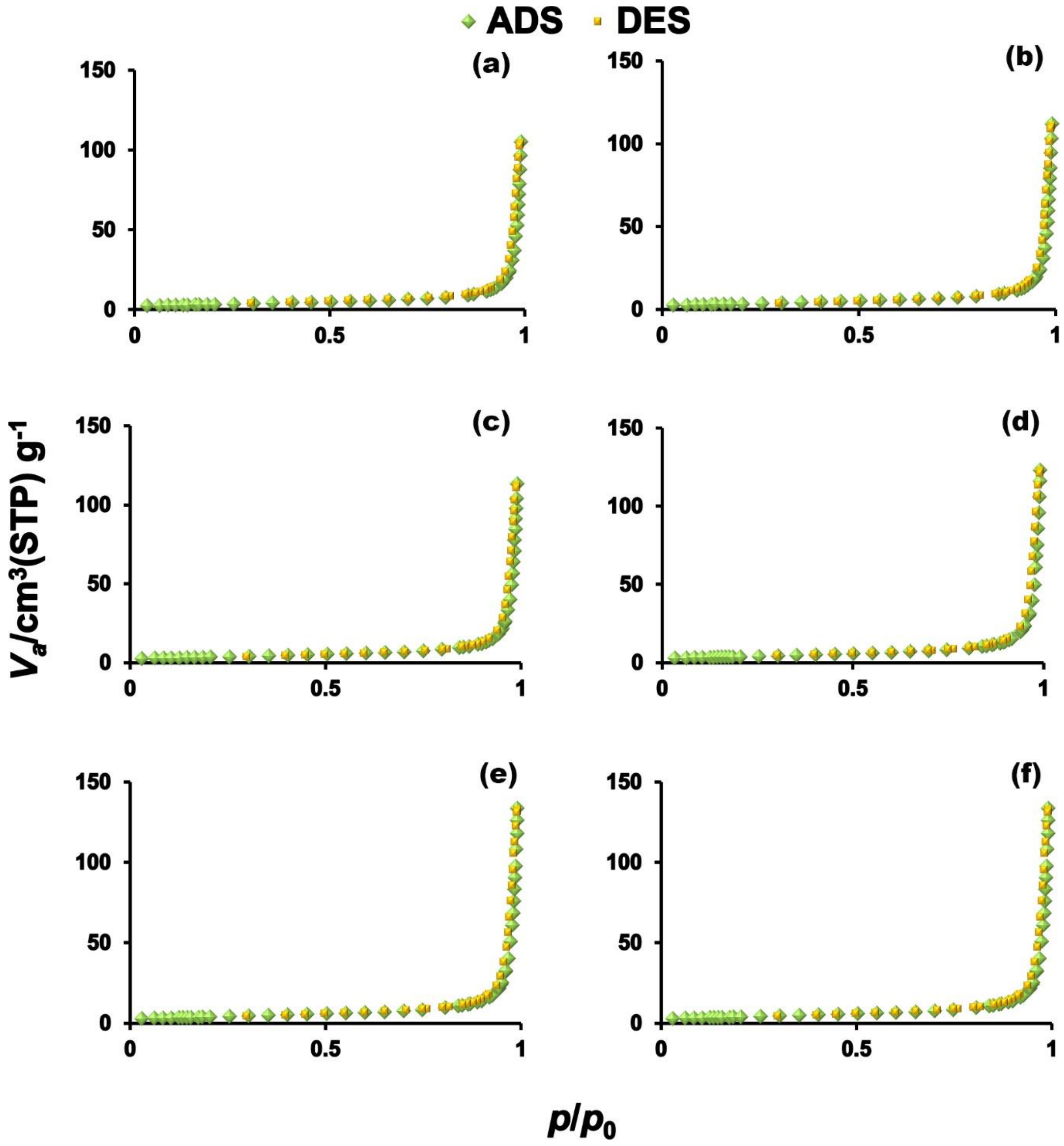

Figure S4. N_2 adsorption-desorption isotherms of **(a)** undoped ZnO **(b)** 1% Ni/ZnO **(c)** 3% Ni/ZnO **(d)** 5% Ni/ZnO **(e)** 7% Ni/ZnO and (**f)** 10% Ni/ZnO nanomaterials.

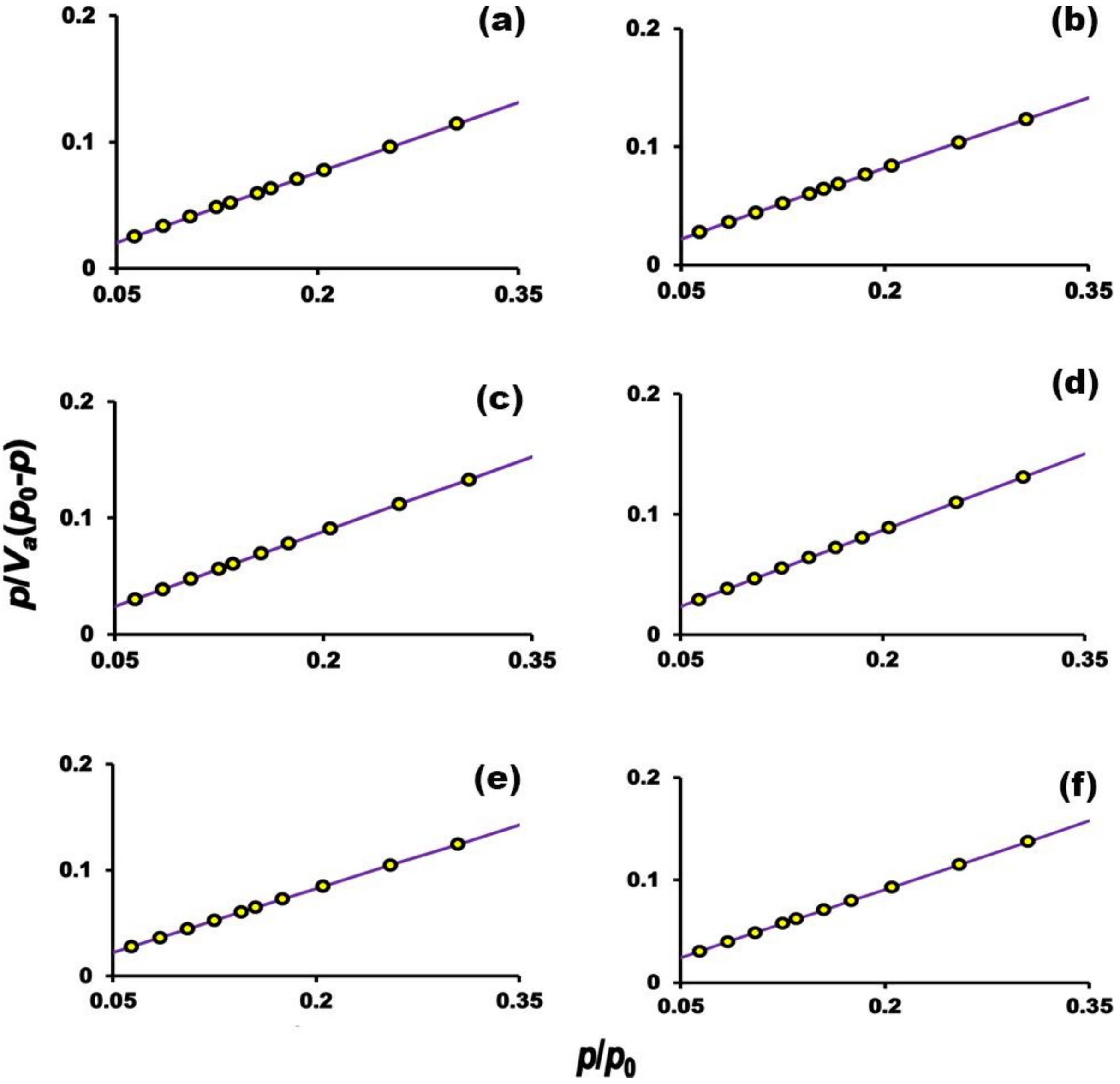

Figure S5. BET plot of **(a)** undoped ZnO **(b)** 1% Ag/ZnO **(c)** 3% Ag/ZnO **(d)** 5% Ag/ZnO **(e)** 7% Ag/ZnO and (**f)** 10% Ag/ZnO nanomaterials.

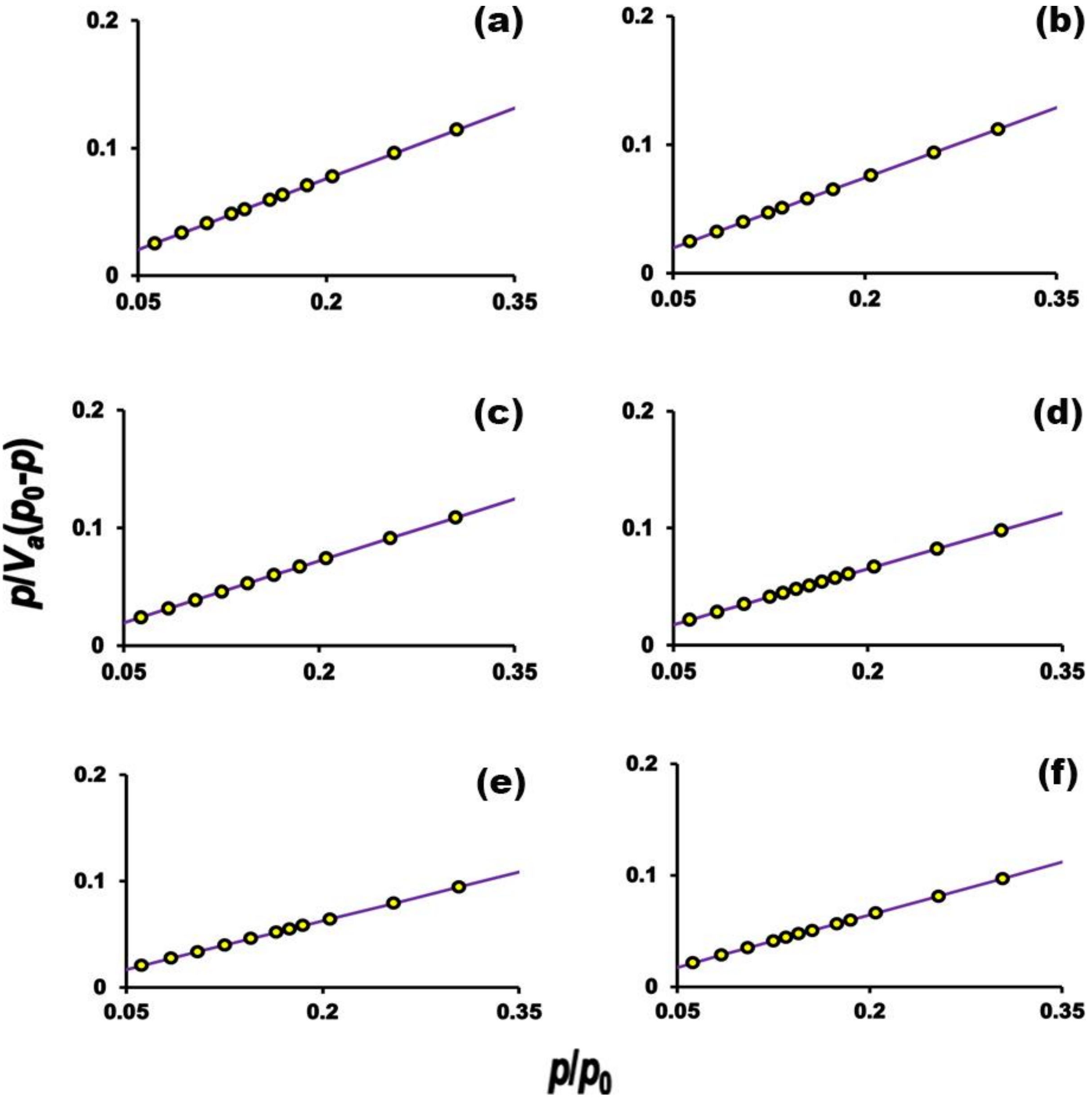

Figure S6. BET plot of **(a)** undoped ZnO **(b)** 1% Ni/ZnO **(c)** 3% Ni/ZnO **(d)** 5% Ni/ZnO **(e)** 7% Ni/ZnO and (**f)** 10% Ni/ZnO nanomaterials.

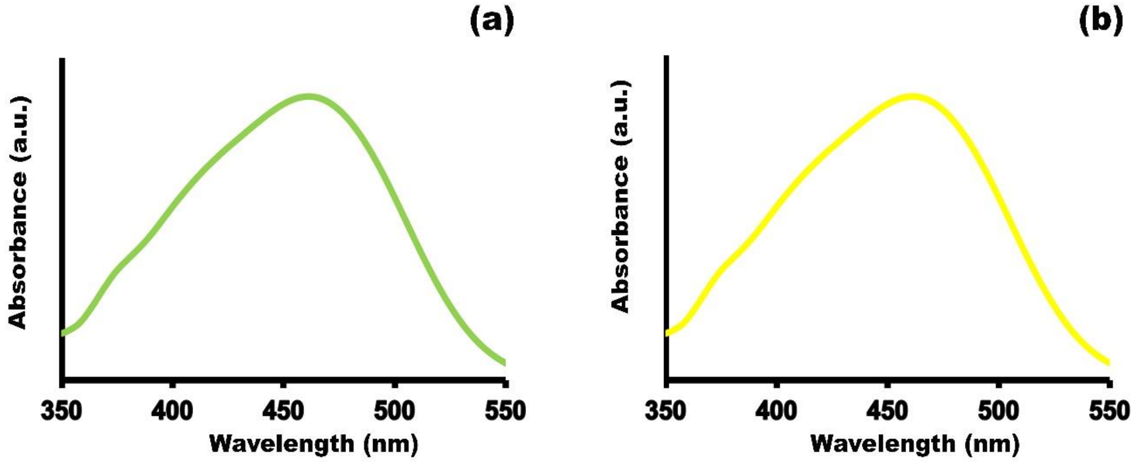

Figure S7. The controlled graph of methyl orange **(a)** without the aids of UV light irradiation and **(b)** without the presence of catalyst for 160 minutes each.

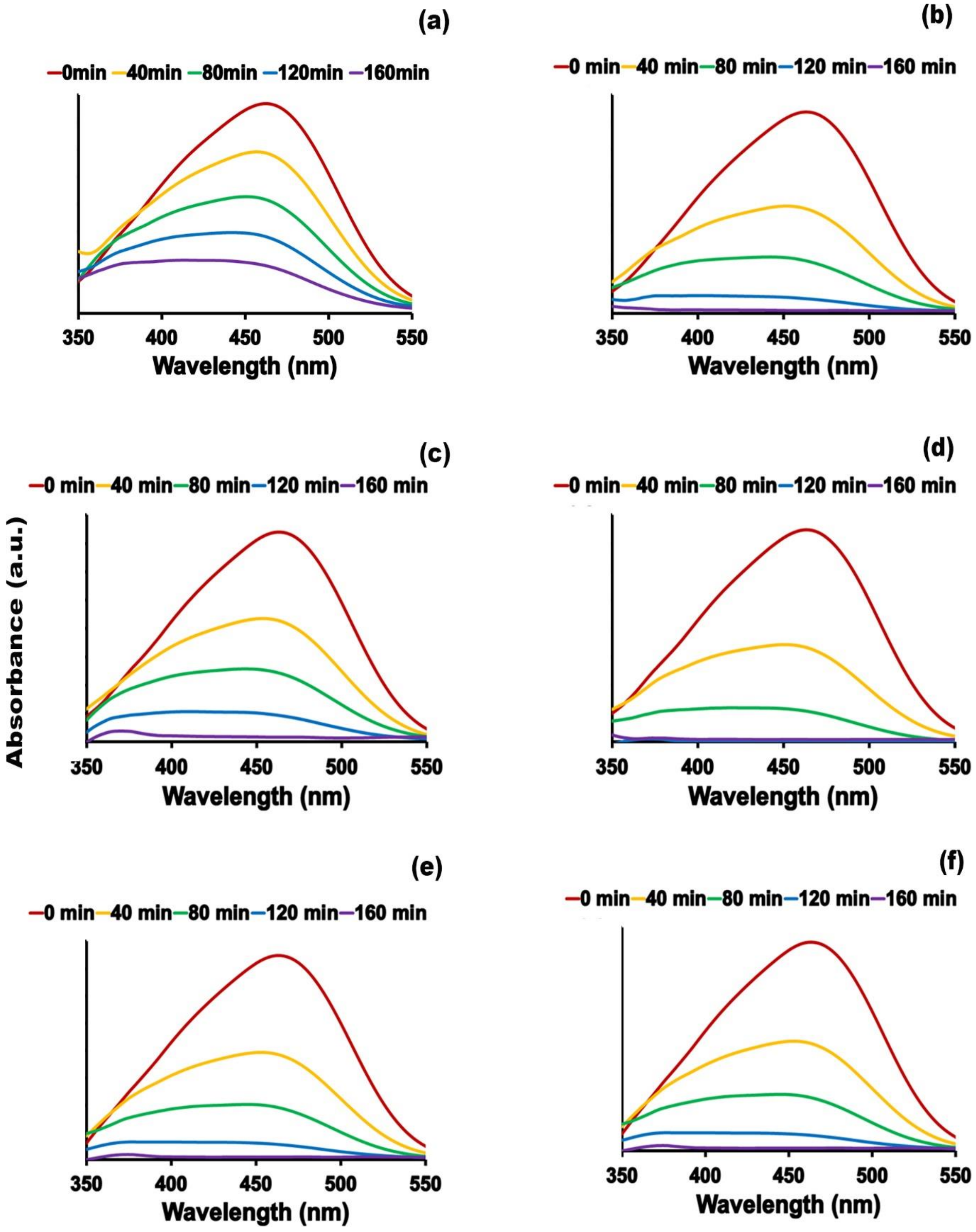

Figure S8. The time dependent absorption spectra of **(a)** undoped ZnO **(b)** 1% Ag/ZnO **(c)** 3% Ag/ZnO **(d)** 5% Ag/ZnO **(e)** 7% Ag/ZnO and **(f)** 10% Ag/ZnO nanomaterials.

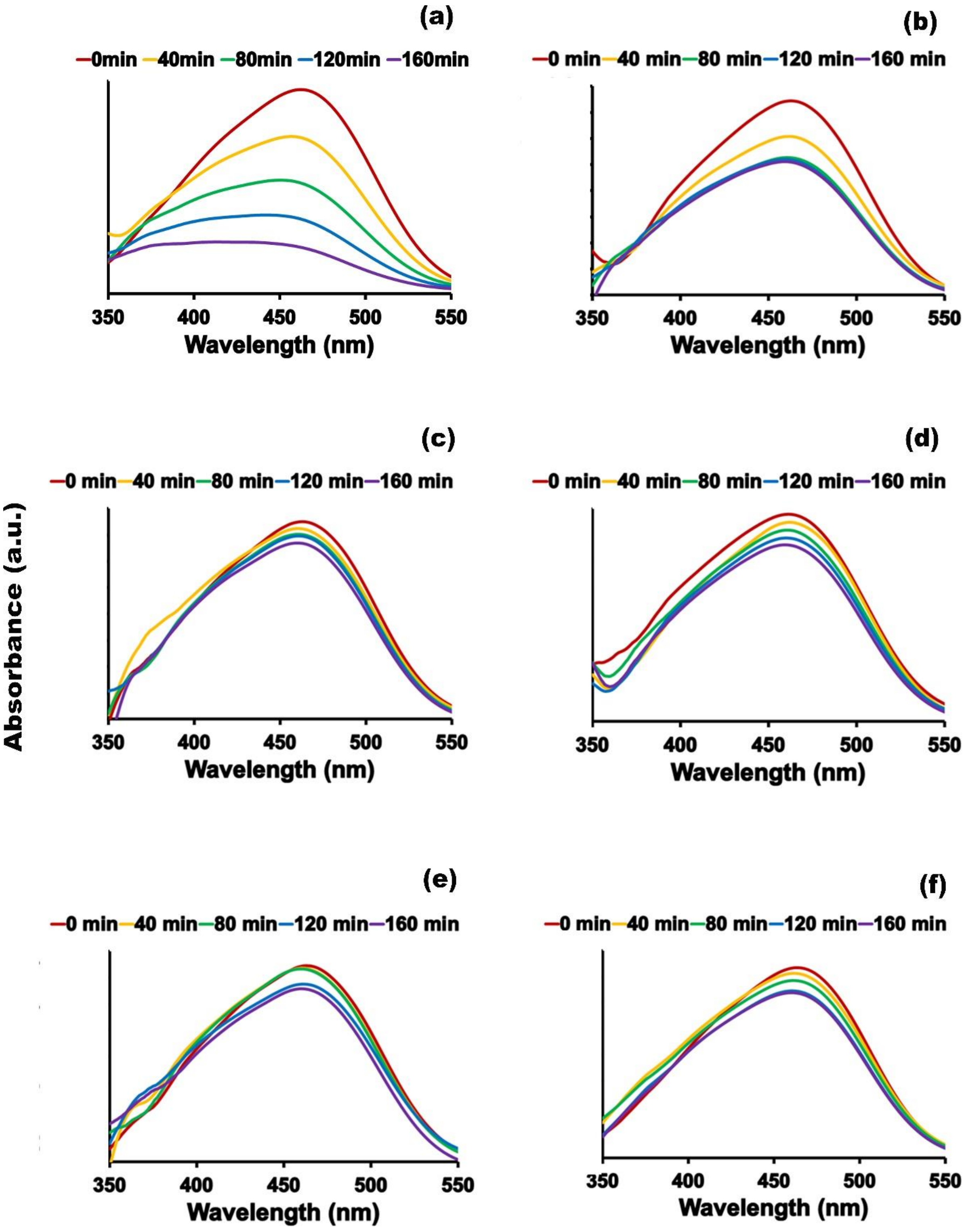

Figure S9. The time dependent absorption spectra of **(a)** undoped ZnO **(b)** 1% Ni/ZnO **(c)** 3% Ni/ZnO **(d)** 5% Ni/ZnO **(e)** 7% Ni/ZnO and **(f)** 10% Ni/ZnO nanomaterials.
